# Focus of attention in musical learning and music performance: a systematic review and discussion of focus instructions and outcome measures

**DOI:** 10.3389/fpsyg.2024.1290596

**Published:** 2024-04-08

**Authors:** Jesper Hohagen, Anna Immerz

**Affiliations:** Freiburg Institute for Musicians’ Medicine, University of Music Freiburg, Medical Center of Albert-Ludwigs-University Freiburg, Freiburg Center for Music Research and Teaching, Freiburg, Germany

**Keywords:** attentional focus, motor learning, musical performance, focus instruction, outcome measures, systematic review

## Abstract

The topic of attentional focus (focus of attention, FOA) in musical learning and performance has recently received increasing interest, as the growing number of empirical studies inspired by the established FOA paradigm in sports by Wulf and colleagues in 1998. The current systematical review aims at collecting, abstracting, and categorizing relevant data to show which kinds of FOA instructions were applied in experimental designs and what kinds of dependent variables were used to measure the effects of FOA instruction on musical performance. The three main inclusion criteria in the selection process were experimental design, detailed descriptions of FOA instructions, and outcome measures (OMs). A systematic search was conducted with a complex search term in four scientific databases in March 2023. For presenting and synthesizing results, we used data collection and an inductive-deductive data categorization. Fifteen studies with a total sample size of 401 participants were included out of 387 records initially identified. We collected 53 different FOA instruction citations from the 15 studies and classified them into 9 FOA subcategories, of which the most applied were *bodily focus* (21%), *sound focus* (15%), and *visual focus* (14%). Selected studies used 63 OMs that were abstracted to 10 different OM categories with *expert ratings* (27%) and *acoustical analysis* (22%) as the most applied dependent variables. Data categorization and abstraction of additional study information show multiple combinations of FOA instructions, OMs, participants’ instruments and expertise, and musical tasks. Finally, studies show no consistent results of superiority of either external or internal or otherwise different FOA considering positive effects on musical performance. Limitations of the review lie in the small study sample, possible criticism of applied eligibility criteria, and subjectivity of data categorization. We propose a research agenda with a more exploratory approach that comprehensively and qualitatively examines the dimensions of musical goals to create a database that could provide a foundation for developing a music-specific FOA model.

## 1 Introduction

The general questions of how we locate our attention while we perform and why we do so play a crucial role in physiological and psychological processes in performances of various everyday life areas as well as in many professional, high-performance domains (e.g., sports, music, dance, etc.). Considering these performance areas in which motor control and motor learning are highly important, we can add many questions that are of special interest to certain research fields, performers, trainers, and educators. Assuming there is a performance effect based on attentional mechanisms, questions arise as to how these processes manifest in different learning or performing situations. What degree of influence does the type of movement, movement task, expertise, pressure, anxiety, state of consciousness, and, finally, instructions have on performance quality and how can we measure it in a domain-specific way? The current systematic review aims to shed light upon some open challenges of focus of attention (FOA) research in general in the music domain by systematically collecting and abstracting data on the two much-discussed aspects of instruction and outcome measures (OMs).

The idea that attentional processes could affect motor performances is not new, nor is it the scientific discourse on it that began at the end of the 19th century and continues today. James (1890, p. 520) describes in chapter XXVI of his book *The Principles about the production of movement* “[…] that we fail of accuracy and certainty in our attainment of the end whenever we are preoccupied with much ideal consciousness of the means.” Moreover, Bliss (1895, p. 55) said in the last sentence of his report, *Investigations in reaction-time and attention,* that it is “[…] a well-known fact that we can perform numerous actions much better when only half attending to them.” From the 1960s, movement and sports research shaped the debate about positive or negative attentional effects on performance from the motor learning perspective. Many of the well-established motor learning theories and concepts refer to development from conscious and highly controlled movements or actions at the beginning of the acquisition of motor skill to a more unconscious and highly free, automatic performance of movements at an expert stage. Meinel (1960) defined this stage as *Variable Verfügbarkeit* (variable availability), in which a performer can detach from movement execution and focus on movement expression (for the role of variability in this regard, see also Bernstein, 1967). Other phase concepts described this as the *autonomous phase* (Fitts and Posner, 1967) that contains automatic mechanisms and the ruggedness of movement execution against external resistances.

### 1.1 Attention under pressure: explicit monitoring and distraction theories

Following this tradition, experimental psychologists of the 1970s and 1980s started to ask and examine whether self-awareness, self-consciousness, and certain attentional processes could aid or detract from performance success (Martens and Landers, 1972; Langer and Imber, 1979), especially in contexts, in which performers are under pressure (Baumeister, 1984; see also Masters, 1992). Later, Beilock and Carr (2001) subsume the explanations for this phenomenon as *explicit monitoring theory* (EMT). The terms *execution focus theory* (see also Beilock and Carr, 2001) and *conscious processing hypothesis* (CPH; Mullen and Hardy, 2000; Wilson et al., 2007) are in line with EMT and highlight that a step-by-step focus on execution degrades performance and this control or self-control disrupts fluency as well as automaticity of movement on the expert level. Although Baumeister (1984) also emphasized the role of anxiety in his concept, other theories put the fear of failing in situations under pressure at the center of their arguments (Wine, 1971; Eysenck, 1979). This *distraction theory* describes processes of involuntary shifts of attention to task-irrelevant information. Another attempt by Eysenck and Calvo (1992) claims that anxiety does not directly impair performance effectiveness but negatively impacts the efficiency of the on-task effort—and further leads to a reduction of processing capacity that results in performance degrading (*processing efficiency theory*, see also Smith et al., 2001; Murray and Janelle, 2003). The first comparative experimental studies from the music and sports domains show that EMT or CPH seems to be a more useful explanation theory for *choking under pressure* processes than the distraction or processing efficiency attempts (Wan and Huon, 2005; Wilson et al., 2007). However, more recent results tended to contradict those insights (Lee and Grafton, 2015; Buchanan et al., 2018; Furuya et al., 2021), whereas other studies seemed to confirm it (Reeves et al., 2007; Gray et al., 2013; Carson and Collins, 2016)—the discussion and research on this topic is ongoing (see also Saikley and Haroush, 2021).

### 1.2 Attentional focus in motor learning and motor performance

In 1998, Gabriele Wulf and colleagues published the results of an experimental study that has received much attention in the last two decades up to the current discourse. Referring to the abovementioned concepts of Baumeister (1984) and Masters (1992), explaining performance degrading under pressure, Wulf et al. (1998) developed an experimental paradigm to examine the effects of different attentional focus instructions on motor learning, independent from the existence of high-pressure situations or anxiety processes. In the first experiment, participants should perform a repetitive skiing-like movement task multiple times with a ski-simulator. The instruction was either “[…] to try to exert force on the outer foot (e. g., the right foot) as long as the platform moved in the respective direction (e. g., to the right side)” (*internal-focus group*) or “to try to exert force on the outer wheels as long as the platform moved in the respective direction” (*external-focus group*; Wulf et al., 1998, p. 172). The results show significantly higher mean amplitude (derived from the platform position data) of movements of the external-focus group compared to the internal-focus group (and a control group that got no specific instruction) in both practice trials and retention tests. A second experiment containing a balance task somehow confirmed these results by showing fewer balance errors in the external-focus group than in the internal-focus group during a retention test after 2 days of practice, in which no further instructions were given. Although a concrete theoretical foundation of that outcome could not be found by the authors at that time, the *common-coding theory* (Prinz, 1997)—describing a common representation of perception and action in the brain—serves as a theoretical framework due to its link to distal events in the form of perception–action coupling mechanisms (an external FOA is a “distal event”). Wulf herself later interpreted that link as insufficient: “Yet, because the theory is rather abstract, it does not specifically predict the differential learning effects of external versus internal attentional foci. It also does not explain any underlying mechanisms of this effect” (Wulf, 2013, p. 91).

Due to the promising results, and despite missing adequate theoretical constructs, Wulf and other researchers applied the paradigm to other movement tasks and could confirm the claimed benefit of an external FOA for motor learning (e.g., Shea and Wulf, 1999; McNevin et al., 2000; Wulf et al., 2000) before formulating the *constrained action hypothesis* (CAH; Wulf et al., 2001a,b). The CAH describes a negative effect chain from (1) focusing consciously on body movement execution or trying to control it, (2) interfering with automatic control motor processes, to (3) a performance degrading or action constraining effect. In addition, or as an expansion, a less beneficial focus on the *self* through an over-evaluation of one’s own actions could enhance the interference effect (*self-invoking trigger hypothesis*, Wulf and Lewthwaite, 2010; McKay et al., 2015). Turning the CAH—which focuses on disadvantageous processes—into an assumption, what kind of FOA could be beneficial for supporting the motor system to be automatic and self-organized, Wulf and colleagues formulate the general instruction advice to focus “[…] on the intended movement effect or task goal” (Wulf and Lewthwaite, 2016, p. 1402). Another hypothesis describes the importance of distance in this regard. The further away a goal appears that refers to external FOA instructions, the greater the effect on motor learning and performance (*distal foci effect hypothesis*; Bell and Hardy, 2009; Duke et al., 2011; McKay and Wulf, 2012; Stambaugh, 2017). Current results from a meta-analysis confirmed the hypothesis that distal-external foci instructions have a more positive impact on motor learning than proximal-external foci instructions (Chua et al., 2021).

An impressive number of experimental studies demonstrate the superiority of the effects of external FOA instructions on motor learning and motor performance. However, there are critical discussions on some aspects of the big picture, e.g., regarding various theoretical issues (Ehrlenspiel and Maurer, 2007; Oudejans et al., 2007; Poolton et al., 2007; Raab, 2007; Peh et al., 2011), methodical questions (Mullen, 2007), and the missing of a theoretical construct that could explain learning benefits of an external FOA (Maurer and Zentgraf, 2007; for an overview, collection of critical commentaries, and responses by Wulf, see the special issue of *Bewegung und Training* [Movement and Training], 2007).

### 1.3 Focus of attention in music

Madsen and Geringer (1990), Geringer and Madsen (1996), Madsen (1997) started a series of studies investigating the focus of attention on different musical elements of musicians and non-musicians while listening to music. Whereas this research is not in line with the attentional effects of focus instructions on motor learning, the results show how different the attentional focus on diverse musical parameters can be in relation to musical expertise or musical stimuli. Wulf and Mornell (2008, see also Mornell, 2007) were the first to investigate the transfer and adaptation of FOA findings from sports to music and dance (see also Guss-West and Wulf, 2016; Mornell and Wulf, 2019). This was followed by an experimental study by Duke et al. (2011) on the effects of attentional instructions on various aspects of solving a short piano task. In their study, participants focused on the fingers, the keys, the hammers, and the sound of the music while playing as part of a repeated measures design. The authors investigated the effect of different FOAs on the evenness of playing movements and showed that non-experts play significantly more consistently in the transfer test when focusing on the hammers and the sound of the music.

Following these results, the FOA paradigm of Wulf et al. (1998), and different explanatory hypotheses (e.g., CAH), some music-related experimental and exploratory studies have been conducted, e.g., in singing (Atkins and Duke, 2013; Atkins, 2017, 2018; Treinkman, 2022a), on the effect in piano playing (Cheng et al., 2011; Lipke-Perry et al., 2022; Jentzsch and Braun, 2023), violin playing (Allingham et al., 2021; Allingham and Wöllner, 2022), wind instrument playing (Stambaugh, 2017, 2019; Williams et al., 2023), or in music education settings (Silvey and Montemayor, 2014; Montemayor et al., 2016; Parsons and Simmons, 2021). The results of these studies vary widely, with some evidence of a positive effect of an external FOA on certain aspects of musical learning and musical performances and some results showing no significant differences between different FOA instructions.

Most of the studies investigating FOA in music predominantly used motor learning and *performance under pressure* models as theoretical underpinnings and transferred those to create an experimental paradigm with a musical task (e.g., Duke et al., 2011; Atkins and Duke, 2013; Atkins, 2017; Stambaugh, 2017; Mornell and Wulf, 2019). Allingham et al. (2021) used an additional music-specific theoretical framework by Jensenius (2007) that presents an *action-sound chain* describing the process from neurological activity in the brain at the start to the production of sound at the end. In more detail, he outlined a paired connection mechanism between the involved performance part and its location area (Brain–Neurological, Muscle–Physiological, Limb–Physical, Instrument–Mechanical, Sound–Acoustical; see Jensenius, 2007, p. 24). In all these parts of the process, a multimodal feedback-loop takes place. This model could be used as an explanation for sound as an external FOA because, at least in the dimension of time, it is the furthest point. Williams et al. (2023, p. 3) provided an attentional focus continuum model for musicians, classifying focus instructions into four main categories from proximal to distal, namely *internal focus*, *external focus*, *distal external focus,* and *very distal external focus*. It is a movement-oriented approach that subsumes insights from the motor learning and music research field but lacks precise sources and theoretical underpinnings regarding what dimension the continuum is grounded on—it could be time, room, and mental capacity. There are only very few approaches to investigating the attention processes of musicians in an explorative and qualitative manner to discover which music-specific aspects condition the direction of an attentional focus. Buma et al. (2015) used a collection of statements from experienced professional musicians to examine different thoughts before and during a performance situation under pressure. The various statements were categorized using cluster analysis and inductively assigned to six focus categories. The individual statements were then evaluated by musicians for their importance and frequency of occurrence during stage performances. The results show that a *musical focus* is mentioned most frequently, but the relevance and application of a musical focus are not considered as important as that of a *focus on physical aspects.* A study by Oudejans et al. (2017), which builds on this, deals with the focus shortly before and after moments of *choking under pressure* and assigns an important significance to a focus on musical aspects here. Another qualitative approach was made by Treinkman (2022a), who examines attentional focus processes in singing by asking more than 200 singers about their foci while practicing and performing. The deductive assignment of the singer’s open statements, where they direct their attention to Wulf’s paradigm of internal vs. external FOA, showed no convincing results regarding which kind of foci were preferred, used, or useful in singing. A qualitative thematic analysis of open-format questionnaires with string players conducted by Lubert et al. (2023) shows four main themes of reported attentional foci during music performance under pressure, that is, navigation of music-related aspects, physical and emotional performance experience, critical thoughts and attempts and control, and quality and dynamic of focus. These explorative studies and inductively performed qualitative analyses are very important for the field due to the differentiated perspective on attentional foci in various musical situations.

### 1.4 Challenges of attentional focus instruction and outcome measures in music

A critical aspect in assessing the effect of attentional instructions in music is the heterogeneity in terms of participants (amateur and professional musicians), instruments (vocals, strings, winds, etc.), and musical tasks or tests (high internal validity and low ecological validity or very application-oriented tasks or music practice interventions). However, the variety of reported verbal attentional instructions and their mainly deductive classification to the internal vs. external FOA categorization by Wulf et al. (1998) lead to difficulties in the interpretation of effects as well as study results. There are some limitations of a dichotomous assignment of instructions for executing a musical task into categories of internal and external FOA (or complementarily possibly far-external and proximal-external FOA). Instructions used in the experiments do not exclusively refer to one movement execution (internal FOA) or one (near or far) movement target (external FOA) as Wulf and colleagues’ paradigm purports (Wulf et al., 1998; see also Wulf, 2013; Wulf and Lewthwaite, 2016). They refer to many different aspects that play a role in making music—namely, body movements, breathing, sound, visual orientation, consistency, communication, visual and auditory imagination, metaphors, musical instruments, physical resistance, creativity, expressivity, musical articulation, etc.—and a music-specific theoretical FOA model that could explain, connect, or differentiate these aspects from another is missing. Recently, Herrebrøden (2023) argued in his critical review that the superiority of external FOA instructions in motor learning experiments in the sports and music domains could alternatively be explained with the direction of instructions on task-relevant information—whereas internal foci instruction often refers to task-irrelevant information.

Finally, the measurement methods used in the experiments are as heterogenic as the various aspects mentioned before. Measuring musical performance is a fundamental problem that plays a major role in a transfer or commonality of sports and musical performance models (“First, we have the problem of measurement,” Schmidt and Lee, 2012, p. 17). In sports and movement research, there is no discussion of the outcomes of gross movement tasks or specific types of sports scoring systems. We can easily measure how high we jump, and we can count baskets, holes, bull’s eyes, or detect errors while trying to reach a task goal (for an overview of outcome measures in the FOA motor learning field, see Chua et al., 2021, p. 622, footnote 2 and Appendix). One of the few exceptions in the FOA research field is expert ratings in gymnastics (Lawrence et al., 2011). In music performance research, the discussion of how to assess musical performances validly and reliably has a long tradition (Saunders, 1993; McPherson and Thompson, 1998; Thompson and Williamon, 2003) and is still up to date (Wesolowski and Wind, 2019a,b; for an overview of different perspectives on the issue from education and research see Brophy, 2019).

### 1.5 Review aims and research questions

Although there was no empirical research on the effects of FOA in music at the time of Wulf and Mornell’s (2008) contribution, the authors formulated implications for music education based on the findings from the field of motor learning: “Teachers will ideally look for verbal instructions that direct attention away from small muscle movements or body, so that automatic motor programs are not disrupted by cognitive interference” (p. 14). Similar deductions are also made based on other results, although the study situation and less evidence do not (yet) provide clear pedagogical or didactical implications for musicians and singers while they practice or perform on stage.

Thus, this review first aims to contribute to a broader discourse in the FOA field by systematically displaying the genesis and actual research situation, mainly in the sports and music domains. Second, we intend to highlight theoretical and methodical challenges and examine to what extent a movement-based model can be transferred to the specifics of musical skill acquisition and music performance. Two of the main questions in this context serve as a framework for the current review and future directions of examining FOA effects in music: What should we focus on and how can we measure it? In more detail and in the context of the present review, we have the following three research questions:

1. How many experimental studies investigate the effects of different attentional focus instructions on learning and performance in the music domain?
2. What kind of FOA instructions, outcome measures, and classifications do they use?
3. Which concrete aspects of FOA research in music should be discussed in the field in the future, and what directions of an application-oriented agenda could there be in music performance research?

## 2 Methods

The present systematical analysis and its methods are strongly oriented to the *PRISMA statement* (Moher et al., 2009) and the updated guidelines for reporting a systematic review (Page et al., 2021). The abstract was written in line with the *PRISMA 2020 for Abstracts checklist* (Page et al., 2021, p. 185). The application of the *PRISMA guidelines* in this systematic review lies in both the methodological process and the structure of illustration by continuously following the *PRISMA 2020 item checklist* (see Page et al., 2021, pp. 183–184). Due to the research topic and main research aims of reviewing FOA instructions and outcome measures, and not effects, the current study did not consider items from the checklist related to meta-analysis recommendations (*11. Study risk of biases*; *12. Effect measures*; *13. Synthesis methods*; *14. Reporting risk of bias*; *15. Certainty assessment*; *18. Risk of bias in studies*; *20. Results of syntheses*; *21. Reporting biases*; *22. Certainty of evidence*, see Page et al., 2021).

A detailed and comprehensive *review protocol*, which can be found in the Supplementary material (Review protocol), contains different tables of datasets to understand the review process better. However, important outcomes referring to the research questions of the current study and additional findings are implemented in the text.

### 2.1 General eligibility criteria

Regarding the whole study selection process recommended by Moher et al. (2009), the eligibility check of reports contains three main levels, i.e., *identification* of records, *screening* of records, and a final, full-text *eligibility* check that includes data collection as well as data abstraction. Beyond singular methodical steps, we defined eight eligibility criteria, which have been reviewed throughout different stages of the selection process. Included studies should meet the following criteria:

- Be published in the English language.
- Be published between February 1998 and March 2023 (due to the first publication of Wulf et al. (1998) presenting the FOA paradigm in movement science).
- Be published in a peer-reviewed journal.
- Refer to the research topic *focus of attention on music* in a broad sense.
- Apply an experimental paradigm referring to Wulf et al. (1998).
- Address the processes of learning or performing a musical skill.
- Contain a precise description of FOA instructions.
- Contain a precise description of outcome measures.

Considering the study selection flow, the first four criteria (a–d) were reviewed in the *screening* phase, whereas the latter four (e–h) were examined during the full-text *eligibility* check.

### 2.2 Information sources and search strategy

To find appropriate reports referring to *FOA in music* as much as possible and guarantee a high degree of transparency, we defined a search term suitable for various scientific search engines and databases. It contains keywords, Boolean operators, truncations, quotation marks, and parentheses. The search with the term (“focus of attention” OR “attentional focus” OR “external focus” OR “internal focus”) AND (music* OR music OR singer OR singing OR voice) was performed using a title/abstract filter in *PubMed*, *SAGE journals*, *Taylor & Francis Online,* and *Web of Science*. In addition, we conducted an open search with the term *“focus of attention” music* in *Google Scholar* and examined the first 300 records (as recommended by Haddaway et al., 2015, who analyzed the procedure, usefulness, and weaknesses of Google Scholar for systematic scientific literature searches in detail). Finally, we scanned the reference lists of representative articles in the field. All actions regarding the systematic search of records were performed by the first author (JH). The search was conducted on 30 March 2023.

### 2.3 Study selection process

After the identification of records and exclusion of duplicates, two reviewers (JH and AI) independently scanned the publishing date, publication type, journal name, record title, and abstract under consideration of the first four eligibility criteria (a–d). If the record did not meet one of the four criteria, it was rated with [EC] for exclusion; otherwise, we assigned the code [TM] for transmission to the next level. In the case of a mismatch rating (a record was rated with [TM] by the first reviewer, but the second reviewer assigned [EC] or vice versa), the records in question were looked at together again and discussed before a decision was made (see the *Review protocol* for review methods, code explanation, and contents of the screening categories, Supplementary material).

Records that met the criteria were transmitted to the final eligibility check, performed by two reviewers (JH and AI) together. At the beginning of this stage, we collected additional basic information about the reports (e.g., *Authors*, *APA citation*, and *DOI*). One report could contain two or more studies (experiments); in such cases, both studies were reviewed. There were no studies published twice, so we did not exclude double-published contributions. Subsequently, we performed a first collection of relevant data in line with the research aim of this review to check the studies under consideration of the latter four eligibility criteria (e–h) and finally, to decide on inclusion or exclusion. Therefore, we created six main data abstraction variables, which are important for the eligibility check, i.e., *type of report*, *research approach*, *research design* (each of these was filled with data by JH and AI, who followed a variable-specific categorization system; see Supplementary material), *FOA instruction description*, *musical task description*, *outcome measure description* (each of these was filled by JH and AI with either [YS] for *reported* or [NO] for *not reported*). In the next step, we checked the four criteria (e–h) for eligibility and finally decided on the inclusion [IC] or exclusion [EC] of the study. In two cases, the decision for selection was made after intensive discussion. Furthermore, the decision about the criteria being fulfilled or missing was distinct.

### 2.4 Data collection, data abstraction, data categorization, and frequency analysis

The included studies were reviewed in more detail with the help of a complex dataset that was divided into five major sections, namely *design*, *participants*, *focus of attention*, *outcome measures,* and *results*. In each of the sections, we created different variables and deductively developed various information categories and an associated code system. The dataset contains four types of variables, namely dichotomous variables (with the codes [YS] for *reported* or [NO] for *not reported*), categorization variables (either with a category system created by us or by the authors of the reviewed studies), and citation variables (with relevant original content from the reviewed studies) or quantitative variables (e.g., sample size). First, this structure provides the basis for an overview of the objective, methods, and outcomes, and second, it lets us focus on the important data to answer the research questions, i.e., (1) the precise wording of FOA instructions and (2) the type of outcome measures trying to assess the effect of those instructions. We either collected FOA instruction classifications (as assigned by the authors of the reviewed studies) or categorized instructions inductive-deductively to give an overview of the field in this regard. Furthermore, we conducted an inductive-deductive categorization of outcome measures, aiming to overview which dependent variables were used in the studies. To display the actual research situation as comprehensively as possible, we finally ran frequency analyses of the most relevant variables in relation to the research questions of this review and displayed percentage distributions accordingly.

## 3 Results

**Figure 1:**
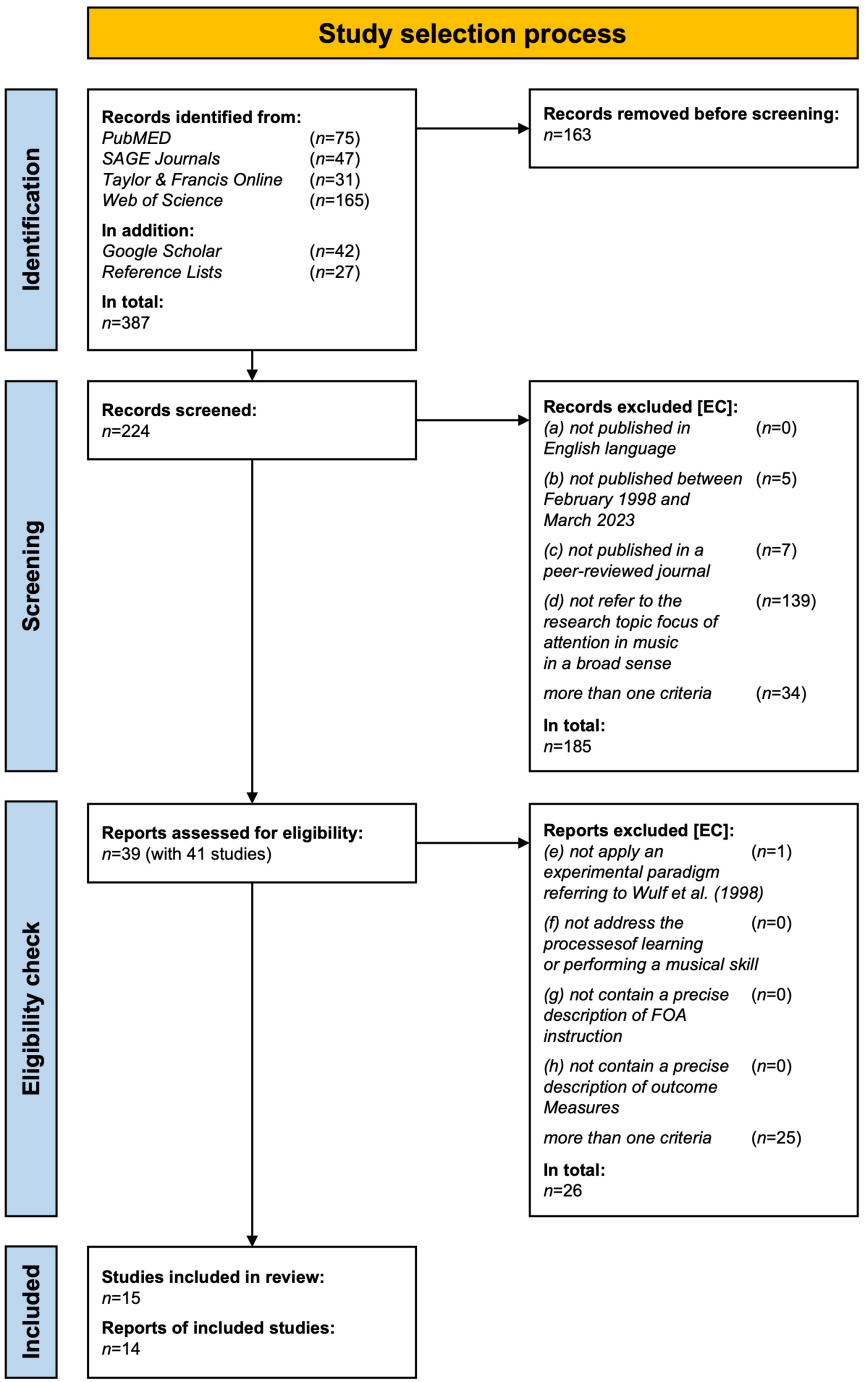
Flow diagram of the study selection process.

The systematic search identified 387 records, of which 163 were duplicates and thus sorted out. The *publishing date*, *title of the journal*, *record title,* and *abstract* of the remaining 224 records were screened by the two reviewers independently. The decision for *exclusion* [EC] or *transmission* [TM] was made with a total agreement rate of 91% (see review protocol, Tab. III, Supplementary material). After discussing critical records and a final agreement about the decision, 185 records (79%) were not transmitted to the final eligibility check. Most records (75%, *n* = 139) were excluded since the contribution did not refer to the research topic FOA in music at all (criteria d), and 4% (*n* = 7) were sorted out because the report was not published in a peer-reviewed journal (c). Five records (3%) were not published between February 1998 and March 2023 (b). The remaining 18% of records (*n* = 34) were not transmitted due to a failure of more than one eligibility criteria (see Figure 1 and Tab. III, Review protocol, Supplementary material). Finally, 39 records passed the screening criteria and were reviewed in detail in the next step.

Within the final full-text eligibility check, we reviewed 39 reports on 41 studies by proofing relevant data in the manuscripts, and we discussed important criteria to decide on study inclusion or exclusion. Finally, 14 reports (36%) with 15 studies were included in the review. One report (Mornell and Wulf, 2019) contains two experiments that should be interpreted as two *studies* following the *Glossary of terms* of the *PRISMA 2020 statement* (see Page et al., 2021, p. 181). Almost all reports (*n* = 25, 96%) were excluded due to the failing of more than one of the four eligibility criteria (e–h). For an overview of the study selection flow, see Figure 1.

After collecting and abstracting relevant data from the studies for the eligibility check, we did another data collection, abstraction, and categorization step for the 15 studies included in the review (see Review protocol, Tab. V, Supplementary material). Many variables containing citations of aims and hypotheses, participant information (e.g., sample size, participants’ instrument, and participants’ expertise), descriptions of musical tasks and material, and detailed information about the results (e.g., post-hoc results) were added to the protocol. Finally, we collected and categorized the important data to answer the review research questions, that is, the correct citations of FOA instructions within the 15 studies and all outcome measures used to measure the effects of experimentally manipulated attentional focus instructions in different experiments. Furthermore, we collected the classifications of FOA instructions by the authors of the original studies and inductive-deductively developed a code system that helps to categorize all focus instructions and assign these categories to the two main FOA classifications by Wulf et al. (1998), that is, *internal* and *external*. The coding and categorization process of dependent variables was conducted with an inductive-deductive attempt to cluster outcome measures into 10 categories (e.g., expert rating, acoustical analysis, and self-evaluation rating).

### 3.1 Attentional focus instruction in music

In total, we collected 53 different experimental FOA instructions. On the first abstraction level, we assigned these instructions to 9 different FOA subcategories inductive-deductively, i.e., either the category is strongly oriented to the original instruction citation or it was assigned by the authors of this review by abstracting on a broader aspect of the FOA field. Fourteen out of 15 included studies designed an experimental paradigm that contains—albeit in different ways—a comparison of different movement (or playing or teaching) instructions made by the experimenter, which refer at least to one *external* and one *internal* aspect of the required task. This fact can be seen as a minimal match across all studies. The study by Williams et al. (2023) serves as an exception due to comparing a practice program that contains external foci with a control group without any FOA instruction. However, the collection, abstraction, and categorization of instruction data show a wide variety of attentional focus instructions with reference to many different goals (see Tables 1, 2).

**Table 1:** Participant information and attentional focus (FOA) instructions of included studies.

Study	Author(s)	Sample size^a^	Expertise	Instrument	Foa instruction^b^ (page)	FOA categorization	
No.	(Year, Experiment)	*N*	Category	Category	Citation	Subcategory^c^	Main category^d^
1	Allingham et al. (2021, Exp I^e^)	32	Both^f^	String	“Focus your attention on the movement in your right arm” (see Table 1, Allingham et al. (2021)	Bodily focus	Internal focus
					“Focus your attention on the sound you produce”	Sound focus	External focus
					“Focus your attention on the resistance of the bow against the string”	Instrumental focus	External focus
2	Allingham and Wöllner (2022, Exp II^e^)	33	Both	String	“Focus your attention on the movement in your right arm” (p. 176)	Bodily focus	Internal focus
					“Focus your attention on the sound you produce”	Sound focus	External focus
					“Focus your attention on the resistance of the bow against the string”	Instrumental focus	External focus
3	Atkins (2017)	22	Experts	Voice	“Focusing their attention to the position of their soft palate” (p. 425)	Bodily focus	Internal focus
					“Focusing their attention on keeping their vibrato steady and consistent”	Technical focus	Internal focus
					“Directing their sound to the top of a tripod placed 18 inches in front of them at mouth height”	Visual focus	External focus
					“Directing their sound to a chair in the center of the performance hall, approximately 24 feet directly in front of the singer and marked with a piece of paper”	Visual focus	External focus
					“Directing their sound to a piece of paper on the back wall of the performance hall approximately 40 feet from the singer and approximately 8 feet above the level of the microphone”	Visual focus	External focus
					“Thinking about filling the room with their sound”	Sound focus	External focus
					“No focus of attention instructions”	Baseline/control	No focus
4	Atkins (2017)	12	Experts	Voice	“Positioning the soft palate” (p. 7)	Bodily focus	Internal focus
					“Keeping their vibrato steady”	Technical focus	Internal focus
					“Directing their sound to the microphone 18 inches in front of them at mouth height”	Visual focus	External focus
					“Directing their sound to a music stand approximately 9 feet across the room at a height of approximately 4 feet”	Visual focus	External focus
					“Directing their sound toward a circle, 4 inches in diameter, drawn on a white board approximately 19 feet across the room and 6 feet above the floor”	Visual focus	External focus
					“No focus of attention instructions were given”	Baseline/control	No focus
5	Atkins and Duke (2013)	30	Novices	Voice	“sing while feeling the vibrations on the throat with either hand” (p. 31)	Bodily focus	Internal focus
					“Sing with the index and middle fingers placed on either side of the nose along the zygomatic arch, which we referred to as the mask, while thinking about directing the sound to the fingers”	Technical focus	Internal focus
					“Sing while thinking about directing the sound to a microphone 18 inches in front of the singer”	Visual focus	External focus
					“Sing while thinking about directing the sound toward a point on the wall, 4 inches in diameter, drawn on the white board approximately 18 feet across the room and 6 feet above the floor”	Visual focus	External focus
					“No focus instructions were given”	Baseline/control	No focus
6	Duke et al. (2011)	16	Both	Piano	“Focus either on their fingers” (p. 48)	Bodily focus	Internal focus
					“The keys”	Instrumental focus	External focus
					“The hammers”	Instrumental focus	External focus
					“Or the sound produced”	Sound focus	External focus
7	Jentzsch and Braun (2023)	49	Both	Piano	“While you perform, I want you to focus on the sounds you are creating” (p. 583)	Sound focus	External focus
					“While you perform, I want you to focus on the movements of your fingers”	Bodily focus	Internal focus
8	Lipke-Perry et al. (2022)	9	Experts	Piano	“Focusing on the fingertips and creating staccato articulation” (p. 4)	Bodily focus	Internal focus
						Technical focus	
					“Focusing on creating the style of the dance”	Metaphorical focus	External focus
					“Focusing on the beat of a metronome set at 144 beats per minute”	Auditory focus	External focus
					“Without any instruction”	Baseline/control	No focus
9	Silvey and Montemayor (2014, Exp I^e^)	32	Novices	Music education	“Focused almost exclusively on ‘internal’ matters related to knowledge of the score and development of an aural image of the music: identified important music lines, such as melody, countermelody, accompaniment, and bass line marked specific music materials in their scores with pencil, pens, or highlighters listened five times to a professional recording of their excerpt while following the score and/or practicing conducting gestures repeatedly sang individual music lines as previously identified notated potential difficulties for individual sections or the ensemble engaged in silent score study” (p. 164)	Other focus	Internal focus
					“Focused their preparations on observable rehearsal behaviors with a minimal amount of time devoted to score study: brief identification of important music lines, such as melody, countermelody, accompaniment, and bass line a general discussion of successful conductor rehearsal behaviors observation of three expert conductors’ successful rehearsal videos self-observation of the previous rehearsal video prior to their upcoming rehearsal for both Sessions 2 and 3 also for Sessions 2 and 3, identification of three goals for improvement after watching their video of the previous rehearsal peer evaluation and discussion of another participant’s rehearsal video, using the RES^g^” (pp. 164–165)	Other focus	External focus
10	Montemayor et al. (2016, Exp II^e^)	32	Novices	Music education	“Participants’ preparation related to knowledge of the score and development of an aural image of the music” (p. 458)	Other focus	Internal focus
					“Participants focused their preparations on observable rehearsal behaviors with a minimal amount of time devoted to score study”	Other focus	External focus
11	Mornell and Wulf (2019, Exp I^h^)	23	Experts	Various instrument categories	“Focus on the precision of their finger movements (or lip movements for singers) and correct notes” (p. 379)	Bodily focus	Internal focus
						Technical focus	
					“Focus on playing for the audience and the expressive sound of the music”	Communicative focus	External focus
						Sound focus	
					“Without specific focus instruction“	Baseline/control	No focus
12	Mornell and Wulf (2019, Exp II^h^)	18	Experts	Various instrument categories	“Focus on the precision of their finger movements (or lip movements for singers) and correct notes” (p. 382)	Bodily focus	Internal focus
						Technical focus	
					“Focus on playing for the audience and the expressive sound of the music”	Communicative focus	External focus
						Sound focus	
					“Play the way they normally did“	Baseline/control	No focus
13	Stambaugh (2017)	30	Both	Wind	“Think about your fingers” (p. 48)	Bodily focus	Internal focus
					“Think about the keys”	Instrumental focus	External focus
					“Think about your sound”	Sound focus	External focus
					“No specific FOA”	Baseline/control	No focus
14	Stambaugh (2019)	56	Novices	Wind	“Think about your fingers, the ones that are moving (woodwind, valved brass group)/think about your hand, the one that is moving (trombone group)” (p. 239)	Bodily focus	Internal focus
					“Think about the sound of your playing“	Sound focus	External focus
					“As accurately as possible and like you heard in the recording”	Baseline/control	No focus
15	Williams et al. (2023)	7	Experts	Wind	“Imagine the phrase/motif you are about to play with as much detail and nuance as you can evoke sing and gesture the phrase/motif dramatically and with detail play the phrase play another version(s) of the phrase repeat the procedure with a new phrase/motif stop if tired, bore avoid analyzing and judgment–focus on the imagined sound avoid mechanical repetition” (see APT^i^, S2)	Other focus	External focus
					“They were instructed to practice in their ‘normal’ way” (p. 5)	Baseline/control	No focus

a Sample size for analysis after drop out or sort out.

b Some citations were displayed without punctuation characters and parentheses; some citations were marginally modified in terms of grammar corrections for suitable illustration; no content was changed.

c Codes were developed and assigned inductive-deductively by interpretation of the FOA instruction content.

d Codes were assigned in two ways: (1) by authors of this review based on links between FOA groups/conditions and FOA categories referring to the FOA paradigm by Wulf (2013) in the original manuscript of the study; (2) by the authors of this review based on an interpretation of original group/condition classification in terms of Wulf’s definitions of an external focus (to focus “[…] on the intended movement effect or task goal,” Wulf and Lewthwaite, 2016, p. 1,402) and an internal focus (“[…] concentration on body movements,” Chua et al., 2021, p. 619).

e Same participants in Experiment 1 and Experiment 2.

f Author(s) did not define or group participants in terms of musical expertise at all or both experts and novices took part in the study.

g Rehearsal Effectiveness Scale (RES; Bergee, 1992; see Silvey and Montemayor (2014) for detailed explanation).

h Different participants in Experiment 1 and Experiment 2.

i Audiation Practice Tool (see Williams et al., 2023).

**Table 2:** Frequencies of FOA instruction categories, experimental groups, or conditions used in included studies.

FOA code	FOA subcategory	Study	N	%
BD	Bodily focus	Duke et al. (2011), Atkins and Duke (2013), Atkins (2017, 2018), Allingham and Wöllner (2022), Stambaugh (2017, 2019, Mornell and Wulf (2019), Exp1&2, Allingham et al. (2021), Lipke-Perry et al. (2022), Jentzsch and Braun (2023)	12	21
SO	Sound focus	Duke et al. (2011), Atkins (2017), Stambaugh (2017, 2019), Allingham et al. (2021), Allingham and Wöllner (2022), Jentzsch and Braun (2023)	9	15
CO	Control/baseline	Atkins and Duke (2013), Atkins (2017, 2018), Stambaugh (2017, 2019), Mornell and Wulf (2019) (Exp1&2), Lipke-Perry et al. (2022), Williams et al. (2023)	9	15
*VS*	Visual focus	Atkins and Duke (2013), Atkins (2017, 2018)	8	14
TC	Technical focus	Atkins and Duke (2013), Atkins (2017, 2018), Mornell and Wulf (2019) (Exp1&2), Lipke-Perry et al. (2022)	6	10
OT	Other focus	Silvey and Montemayor (2014), Montemayor et al. (2016), Williams et al. (2023)	5	9
IS	Instrumental focus	Duke et al. (2011), Stambaugh (2017), Allingham et al. (2021), Allingham and Wöllner (2022)	5	9
*CF*	Communicative focus	Mornell and Wulf (2019) (Exp1&2)	2	3
AF	Auditory focus	Lipke-Perry et al. (2022)	1	2
MP	Metaphorical focus	Lipke-Perry et al. (2022)	1	2
Total			58	100

The most frequently used instructions refer to an attentional focus on the body (*n* = 12, 21%), a FOA on sound (*n* = 9, 16%), and a control condition or control group, in which generally no specific focus instructions were given (*n* = 9, 16%). Instructions referring to a visual focus were solely used in studies with singers and investigating FOA instructions on different singing tasks (Atkins and Duke, 2013; Atkins, 2017, 2018). The reason for this specificity lies in the behavior that singers do not have naturally to visually focus their instruments, as they are hidden inside the body, so they can adopt an attentional focus while visualizing different focal points in their environment. An instrumental focus was instructed in 4 of the 15 studies reviewed. Furthermore, there are a few experimental FOA instructions that we could not assign reasonably to one of the other 9 subcategories because they were part of a whole practice (Williams et al., 2023) or education program (Silvey and Montemayor, 2014; Montemayor et al., 2016) and their content between and within the program was very different. That is why they were subsumed in the category *Other focus* (see Table 2).

Those 10 subcategories were assigned to three main categories of a category system that is oriented on the original experimental paradigm of Wulf et al. (1998); see also (Wulf, 2013) consisting of FOA instructions either as internal, external, neutral, or with no specific focus. In the current review, we abstracted each of the FOA instructions used and 10 subcategories to one of these three main categories based on either a note for a link in the original manuscript of the study or due to an interpretation of the original group/condition classification in terms of Wulf’s definitions of an external focus (to focus “[…] on the intended movement effect or task goal,” Wulf and Lewthwaite, 2016, p. 1,402) and an internal focus (“[…] concentration on body movements,” Chua et al., 2021, p. 619). Within the 15 studies reviewed, 27 (51%) external foci have been used, 32% of all FOA instructions refer to internal attentional processes (*n* = 17), and 9 instructions had no special focus and served as control or baseline conditions (17%, see Table 3).

**Table 3:** Frequencies of FOA instruction categories referring to the paradigm by Wulf (2013).

FOA code (wulf)	FOA category (Wulf)	N	%
IF	Internal focus	17	32
EF	External focus	27	51
NF	No focus, baseline, control condition, control group	9	17
Total		53^a^	100

a Total frequency of foci in reference to Wulf (2013) differs from the number of subcategories (N = 58) since five instructions got two assignments of subcategories, which both refer to the same categories by Wulf.

For internal focus instructions used by experimenters and authors of the studies, the wording appears often sharp, precise, and goal-oriented with a clear link to body parts or body motions, e.g., “focus on your fingers” (Duke et al., 2011), “focus on your right arm” (Allingham et al., 2021), “focus on the precision of their finger movements” (Mornell and Wulf, 2019), or “focus on your soft-palate” (Atkins, 2017). In the included studies, 21% of all instructions refer to the body (*n* = 12, see Table 2). On the other hand, there are internal FOA instructions relating to different dimensions, such as a type of auditory imagery (“[…] and development of an aural image of the music,” Silvey and Montemayor, 2014) or reference to notes or the score (“[…] and correct notes,” Mornell and Wulf, 2019). Some authors classify a focus on technical performance aspects as an internal FOA, e.g., “[…] keeping their vibrato steady and consistent” (Atkins, 2017) or “[…] and creating staccato articulation” (Lipke-Perry et al., 2022), even if the words refer to outcomes that could be interpreted by definition (see Wulf, 2013) as movement goals – that is, in reference to the abovementioned instructions, move in a certain manner *to* sing consistently or *to* create staccato.

Within the package of instruction wordings of the 15 included studies referring to external attentional foci in music (*n* = 27), the interpretation, abstraction, or classification is challenging, at least due to the variety of musical tasks, materials, participants’ experiences, and participants’ instruments. However, one crucial aspect is the interpretation of *sound* as the central goal of musical movements or musical tasks in equivalence or as a modification of the definition of external FOA in motor learning, *focusing on the movement effect* (see Wulf, 2013). This is supported by the percentage of instructions categorized as *sound focus* (*n* = 9, 33% of all external FOA instructions). A further subcategory of special relevance for research in singing is classified as *visual focus* (*n* = 8, 30% of external FOA instructions). An important difference compared to *sound* as a movement effect is the action dimension of visualizing a certain point (either near or far away) in the room (see Atkins and Duke, 2013; Atkins, 2017, 2018). Adopting a visual focus while making music is not a focus on a movement effect or movement goal; it can be seen as a supporting moderator between movement execution and sound to optimize sound. Obviously, concentrating on a visual task during a music performance is easier when musicians are not physiologically and perceptually tied because hand–eye coordination is not essential, as for singers.

Another important type of external FOA can be described as an *instrumental focus* in general (*n* = 5, 19% of external FOA instructions). Looking back at the original experimental design and experimentally manipulated instruction in the ski-simulator study by Wulf et al. (1998), an attentional instruction focusing on the musical instrument (or maybe certain aspects of voice as the pendant for singers) has the biggest theoretical overlap to *the* original external instructions in motor learning, focusing on not the feet but the wheels of the ski-simulator platform. Allingham et al. (2021); see also (Allingham and Wöllner, 2022) used the term *somatic focus* to depict the importance of physical resistance and the tactile sensory feedback while focusing on the instrument. Duke et al. (2011) and Stambaugh (2017) also used the instrumental focus on keys to find differences between concentrating on essential parts of the piano or the wind instrument and concentrating on the sounds that arise through actuating these essential parts.

### 3.2 Outcomes measures in FOA studies in the music domain

Across all included studies, we collected 63 descriptions of dependent variables to measure the outcomes and, thus, the effects of experimentally manipulated attentional focus instructions on the performance of a musical task. Thereby, the distribution of used outcome measures per study is very heterogeneous and reaches from 1 (Stambaugh, 2019; *Temporal evenness*) to 14 (Allingham et al., 2021; 3 different OM categories). A detailed collection of OM and other relevant information can be overviewed in Table 4. An inductive-deductive data categorization of all OM descriptions of the original manuscripts resulted in 9 different OM categories (see Table 5), of which *expert ratings* (EXR) were used most to measure FOA effects (*n* = 17; 27%). In total, 7 out of 15 studies used EXR. Furthermore, another 4 studies used different kinds of 14 *acoustical analyses* of recorded performances as dependent measures (ACU; 22%), e.g., roughness, spectral centroid, formant frequencies, or harmonic-to-noise ratio (see Table 5). *Electromyography analysis* (EMG) for measuring the effects of FOA on muscle activity or muscle energy—and therefore on movement efficiency—was solely conducted by two studies of the same research group (Allingham et al., 2021; Allingham and Wöllner, 2022). However, six different EMG measures were applied (10%), the same amount as for *self-evaluation ratings* (SER; 10%) and *error detection* (ERD; 10%). One study used another’s evaluation ratings (OER; 3%; Silvey and Montemayor, 2014), that is, an evaluation rating of performance conducted neither by an expert nor by oneself. Furthermore, one study measured FOA effects with the help of movement analysis (MVA) and used five different movement parameters (8% of all OM).

**Table 4:** Musical tasks, outcome measures (OMs), and results of included studies.

Study	Author(S)	Research design (factors)	Musical task and material	Task paradigm	Outcome measures	OM category	Results
No.	(Year, Experiment)	Category	Paraphrase	Category	Paraphrase	Category as code	FOA sub category (</>/=)^a^^b^
1	Allingham et al. (2021, Exp I^c^)	Mixed Design (Between-subject factors [NV,EX] x Within-subject factors [BD,SO,IS)	*General bowing task:* Bow string task (4x) on the open A-string in response to a metronome *Further task-specific instruction/requirements:* Playing in time with metronome, playing with a good, consistent sound and avoiding scratching sounds	Performance paradigm	Mean spectral centroid of audio signal	ACU	**IS >^d^** BD/IS = SO/BD = SO
					SD spectral centroid of audio signal	ACU	n.s.
					M roughness of audio signal	ACU	n.s.
					SD roughness of audio signal	ACU	n.s.
					M root mean square of audio signal	ACU	n.s.
					SD root mean square of audio signal	ACU	n.s.
					M bow contact point	MVA	n.s.
					SD bow contact point	MVA	**IS <** SO/IS = BD/BD = SO
					Scroll sway (freedom of motion)	MVA	**IS >** BD^e^/IS = SO/BD = SO
					M bow acceleration	MVA	n.s.
					SD bow acceleration	MVA	n.s.
					Deltoid muscle activity	EMG	**IS <** BD/IS = SO/BD = SO
					Tricep muscle activity	EMG	n.s.
					Bicep muscle activity	EMG	n.s.
2	Allingham and Wöllner (2022, Exp II^c^)	Mixed Design (Between-subject factors [NV,EX] x Within-subject factors [BD,SO,IS)	*General bowing task:* Slow motion bow sound production task *Further task-specific instruction/requirements:* Nuanced, slow motor control skills, no lift of the bow, no changing of direction	Performance paradigm	Number of clicks	ACU	n.s.
					Number of errors	ACU	**IS <** BD^e^/IS = SO/BD = SO
					Deltoid muscle activity	EMG	n.s.
					Tricep muscle activity	EMG	**IS <** BD/IS = SO/BD = SO
					Bicep muscle activity	EMG	n.s.
3	Atkins (2017)	Within-subject design (BD[SP],TC[VI],VS[TN],VS[CM],VS[PO], FL[SO],CO)	*Different singing tasks:* Singing a three-note [α] vowel pattern (low) three-note [α] vowel pattern (high) 1st full phrase of “My Country ’Tis of Thee’” 1st or 2nd phrase of a song by choice	Performance paradigm	Ring	EXR	^f^(1) **FL >** CO&SP&VI&TN&CM&PO/**PO >** CO&VI&TN ^f^(2) **FL >** CO&SP&VI&TN&CM&PO/**PO >** CO&VI&TN ^f^(3) **FL >** CO&SP&VI&TN&CM/**SP >** CO/**PO >** CO&VI ^f^(4) **FL >** CO&SP&VI&TN&CM&PO/**PO >** CO
					Evenness	EXR	n/a
					Vibrato	EXR	n/a
					Freedom	EXR	n/a
					Intonation	EXR	n/a
					Color	EXR	n/a
					Overall	EXR	^f^(1) **FL >** CO&TN ^f^(2) **FL >** CO&TN ^f^(3) **PO >** CO&CM/**Fl >** CO ^f^(4) **FL >** SP
4	Atkins (2018)	Within-subject design (BD[SP],TC[VI],VS[MN],VS[SM],VS[PO],CO)	*Different singing tasks:* Singing a three-note [α] vowel pattern 1st or 2nd phrase of a song by choice	Performance paradigm	Overall Assessment	EXR	(1) n/a^g^ (2) n/a^g^
					Mean harmonic-to-noise ratio	ACU	(1) n.s. (2) n.s.
					Intensity	ACU	^f^(1) **PO >** CO/**SM >** VI/**SP >** VI (2) n.s.
					Formant frequencies (F1-F5)	ACU	(1) n.s. (2) n.s.
5	Atkins and Duke (2013)	Within-subject design (BD[TH],TC[MA],VS[MN],VS[PO],CO)	*General singing task:* Singing a three-note [α] vowel pattern	Performance paradigm	Overall ranking (1st–5th)	EXR	n/a
					Mean frequency (Hz)	ACU	n/a
					Formant frequencies	ACU	n/a
					Harmonic-to-noise ratio	ACU	n/a
6	Duke et al. (2011)	Within-subject design (BD[FI],IS[KY],IS[HA],SO)	*General piano task:* Playing a 13-note sequence composed of alternating sixteenth notes using the index and ring fingers of the right hand (for acquisition and retention, slightly different for transfer task) *Further task-specific instruction/requirements:* Playing as quickly and evenly as possible	Learning paradigm	Temporal evenness (IOI SD)	PHY	^h^(a) n/a (b) n/a (c) n/a (d) n/a (R) n.s ^f^(T) **HA <** BD/**SO <** BD/BD = KY/HA = SO
					Loudness evenness (KV SD)	PHY	n/a
7	Jentzsch and Braun (2023)	Mixed design (Between-subject factors [EX,IM] x Within-subject factors [SO,BD])	*General piano task:* Playing first 24 bars of *J. S. Bach’s “Little Prelude in D Minor” BWV 935 (Score on Line—Digital Sheet Music Library—partitions de musique classique, 2020*) *Further task-specific instruction/requirements:* No stress, should not be perfect	Performance paradigm	Pitch errors	ERD	**SO <** BD
					Hesitations	ERD	n.s.
					Note corrections	ERD	**SO <** BD
					Deletions	ERD	n.s.
8	Lipke-Perry et al. (2022)	Within-subject design (BD/TC, OT, AF, CO)	*General piano task:* Playing Bartók’s Romanian Folk Dance, Sz. 56, No. 2 *Further task-specific instruction/requirements:* Articulation and pedaling instruction were in the score, no other preparatory instruction	Performance paradigm	Pedal Performance Z-Score^i^:	PHY	n/a
					Expert Listener Rating of Performances	EXR	n/a
9	Silvey and Montemayor (2014), Exp I^c^)	Between-subject design (OT[AI], OT[RB])	*General teaching task:* Leading an ensemble in a series of three 6-minute rehearsals on their assigned excerpt. Materials were from Volumes 1 and 2 of Teaching Music Through Performance in Band (Miles, 1997-1998)	Performance paradigm	Conductor self-evaluation of teaching	SER	n.s.
					Conductor evaluation of ensemble	SER	n.s.
					Ensemble eval. of conductor effectiveness	OER	n.s.
					Ensemble eval. of conductor score knowledge	OER	n.s.
					Panel audio eval. of ensemble performance	EXR	n.s.
10	Montemayor et al. (2016, Exp II^c^)	Between-subject design (OT[AI], OT[RB])	See Study 9 (Silvey and Montemayor (2014), Exp I)	Performance paradigm	Frequencies of teachers’ verbal behaviors, assigned to 16 musical var. (see Table S2; Montemayor et al., 2016)	FQA	Sign. difference for 1 out of 16 variables: Balance/blend (**AI >** RB)/all other var.: n.s.
					Frequencies of teachers’ verbal behaviours, assigned to 7 teaching var.	FQA	Sign. difference for 1 out of 8 variables: Positive feedback/specific (**RB >** AI)/all other var.: n.s.
					Clarity of gesture	EXR	n.s.
					Expression	EXR	n.s.
11	Mornell and Wulf (2019, Exp I^j^)	Within-subject design (BD/TC, SO/CF, CO)	*General performing task:* Playing music/singing a song of their choice of approximately 3-minute duration, that they had performed in concert	Performance paradigm	Technical precision	EXR	n.s.
					Musical expression	EXR	**SO/CF >** BD/TC/**SO/CF >** CO/BD/TC = CO
12	Mornell and Wulf (2019, Exp II^j^)	Within-subject design (BD/TC, SO/CF, CO)	See Study 11 (Mornell and Wulf, 2019, Exp I)	Performance paradigm	Technical Score (mean of 5 items)	EXR	**SO/CF >** BD/TC/SO/CF = CO/BD/TC = CO
					Musicality Score (mean of 5 items)	EXR	**SO/CF >** BD/TC/**SO/CF >** CO/BD/TC = CO
13	Stambaugh (2017)	Mixed design (Between-subject factors [NV,EX] x Within-subject factors [BD,IS,SO,CO)	*General wind task:* Playing a 9-note sequence composed of alternating eights-notes using the index and ring fingers of the right hand (for acquisition and retention, slightly different for transfer task) *Further task-specific instruction/requirements:* Playing as evenly and accurately as possible, coordination between fingers on both hands, breathing, and tonguing	Learning paradigm	Temporal evenness (IOI SD)	PHY	*Novices:* (A) **BD >** CO/**SO >** CO/all other var.: n.s. (R) n.s. (T) n.s. *Experts:* (A) **BD >** CO/**SO >** CO/**BD >** IS/all other var.: n.s. (R) n.s. (T) n.s.
					Pitch error (accuracy)	ERD	*Novices:* (A) **BD >** CO/**SO >** CO/**IS >** CO/all other var.: n.s. (R) n.s. (T) n.s. *Experts:* (A) **BD >** CO/**SO >** CO/**IS >** CO/all other var.: n.s. (R) **BD >** CO/**SO >** CO/**BD >** IS (T) n.s.
14	Stambaugh (2019)	Within-subject design (BD, SO, CO)	*General wind task:* Listening to audio file and subsequently playing each of three different 7-note patterns of alternating eights-note *Further task-specific instruction/requirements:* Playing as evenly and accurately as possible	Learning paradigm	Temporal evenness (IOI SD)	PHY	n/a
15	Williams et al. (2023)	Within-subject design (OT, CO)	*General trumpet task:* Playing unfamiliar excerpts from baroque trumpet literature (J. S. Bach and C. P. E. Bach), participants had to practice 1 test piece three times a day 5 minutes for three days (control phase) in a “normal” way; same procedure in intervention phase, but with following APT (see Table 1)	Performance paradigm/Learning paradigm	Pitch error (accuracy)	ERD	**OT >** CO
					Confidence	SER	n.s.
					Motivation	SER	n.s.
					Engagement	SER	n/a
					Self-efficacy	SER	n.s.

(a) = 1st training block, (A) = Acquisition block, ACU = Acoustical analysis, AF = Auditory focus, AI = (focus on) Aural image, APT = Audiation Practice Tool (see Williams et al., 2023), (b) = 2nd training block, BD = Bodily focus, (c) = 3rd training block, CF = Communicative focus, CM = (focus on) Chair-middle, CO = No focus/baseline/control condition/control group, (d) = 4th training block, EMG = Electromyography analysis, ERD = Error detection, eval. = evaluation, EX = Experts/professionals/advanced players, Exp = Experiment, EXR = Expert rating, F = Formant, FI = (focus on) Fingers, FL = Fill (the room with sound), FQA = Frequency analysis, HA = (focus on) Hammers, HZ = Hertz, IM = Intermediate players, IOI = Inter onset interval, IS = Instrumental focus, KV = Keystroke velocity, KY = (focus on) Keys, M = Mean, MA = Mask (focus on the fingers on the nose), MN = (focus on) Microphone-near, MVA = Movement analysis, n/a = Results not available or not reported, No. = Number, NV = Novices, amateurs, n.s. = Not significant, OER = Others’ evaluation rating (not self, not experts), OT = Other focus, PHY = Physical analysis, PO = (focus on) Point-far, (R) = Retention test(s), RB = (focus on) Rehearsal behavior, SD = Standard deviation, SER = Self-evaluation rating, SM = (focus on) Stand-middle, SO = Sound focus, SP = (focus on) Soft-palate, (T) = Transfer test(s), TC = Technical focus, TH = (focus on vibrations in the) Throat, TN = (focus on) Tripod-near, var. = variables, VI = (focus on) Vibrato, VS = Visual focus.

a if *post-hoc* results are available.

b if more than one subcategory was implemented in an experimental paradigm (e.g., three times Visual focus, see Atkins, 2017), we used more specific codes that distinguish the different instructions referring to the same category (e.g., TN for Tripod-near, CM for Chair-middle, PF for Point-far, which all refer to Visual focus, see Atkins, 2017).

c Same participants in Experiment 1 and Experiment 2.

d FOA subcategories in bold were superior to those not bold-faced.

e Just for experts.

f Just significant post-hoc results displayed, all other post-hoc comparisons were not significant.

g No inference statistical analysis, just quantification of qualitative statements.

h Just for less-skilled pianists (N = 12).

I See Lipke-Perry et al. (2022) for a detailed calculation of z-score.

j Different participants in Experiment 1 and Experiment 2.

**Table 5:** Frequencies of outcome measures (OMs) categories used in included studies.

OM code	Outcome measures category	Study	N	%
EXR	Expert rating	Atkins and Duke (2013), Silvey and Montemayor (2014), Atkins (2017, 2018), Lipke-Perry et al. (2022)	17	27
ACU	Acoustical analysis	Atkins and Duke (2013), Atkins (2018), Allingham et al. (2021), Allingham and Wöllner (2022)	14	22
EMG	Electromyography analysis	Allingham et al. (2021), Allingham and Wöllner (2022)	6	10
ERD	Error detection	Stambaugh (2017), Jentzsch and Braun (2023), Williams et al. (2023)	6	10
SER	Self-evaluation rating	Silvey and Montemayor (2014), Williams et al. (2023)	6	10
MVA	Movement analysis	Allingham et al. (2021)	5	8
PHY	Physical analysis	Duke et al. (2011), Stambaugh (2017, 2019), Lipke-Perry et al. (2022)	5	8
FQA	Frequency analysis	Montemayor et al. (2016)	2	3
OER	Others’ evaluation ratings	Silvey and Montemayor (2014)	2	3
Total			63	100^a^

a Total percentage has been marginally adjusted due to rounding up of singular percentage values.

In general, the total frequency of different OM used in FOA studies in the music domain is very high considering the small number of experimental studies in the field (on average, 4.2 OM per study, *sd* = 3.2). One explanation of this result could lie in the explorative character of the included studies, although all studies applied an experimental design with a relatively fixed paradigm. Due to the tenuous research situation in this regard and the lack of clear results yet, a one-sided *focus* on one OM may not be adequate for the explorative aim of most of the research groups.

### 3.3 Further outcomes

In addition to the main research aim, to depict the actual research work in terms of FOA instructions and OM, a few other important outcomes emerged based on the full-text analysis and the data abstraction process. In line with the argument of the explorative character of the studies, frequency analysis showed that just 4 studies out of 15, to the best of our knowledge, formulated a clearly directed hypothesis (Mornell and Wulf, 2019, Exp1&2; Allingham et al., 2021; Allingham and Wöllner, 2022), in which the type of FOA was assumed to be superior regarding the music performance or music learning effect.

In addition to the illustration of FOA instructions, FOA sub, and main categories, Table 1 depicts the sample size, participants’ expertise (experts, novices, and both), and participants’ instrument category. Across all included studies, the sample size ranges from 7 to 52, with a total sample size of 401 and an average of 27.7 participants per study (*sd* = 13.8). In terms of participants’ expertise, the frequency distribution is homogenous, with six studies investigating experts, five studies that defined either two groups of experts and novices or no distinction at all (both), and four studies solely with participants and authors defined as novices or amateurs. As expertise plays a big role in the discussion of FOA effects in sports or motor learning (see *Introduction* and Singh and Wulf, 2020), this differentiation was discussed in many of the included studies (e.g., Stambaugh, 2019; Allingham and Wöllner, 2022; Jentzsch and Braun, 2023). Regarding the participants’ instruments category, which is strongly correlated with the type of musical task within the experimental design (see Table 4), all classical instrumental groups (wind, string, piano, and voice) are present in the included studies except for percussion instruments. However, there are two additional studies that examined music educational skills in ensemble teaching (Silvey and Montemayor, 2014; Montemayor et al., 2016) and two studies that apply the same task to various instrumental groups (Mornell and Wulf, 2019, Exp1 & 2).

Table 4 shows the research design (within-subject, between-subject, and mixed), musical tasks, the task paradigm (performance or learning paradigm) of each study, and a summarized display of post-hoc results for each OM. There are two included studies investigating FOA effects without a within-subject factor and, hence, a between-subject design containing one *internal* FOA group and one *external* FOA group (Silvey and Montemayor, 2014; Montemayor et al., 2016). In addition to that, we found three studies with a mixed design, all of them with the experimental and control conditions as within-subject factor and expertise as between-subject factor (Allingham et al., 2021; Allingham and Wöllner, 2022; Jentzsch and Braun, 2023). Musical tasks are very particular, each of them specially adjusted for the participants’ instrument, research design, and performance paradigm. The two studies that applied a traditional learning paradigm (Duke et al., 2011; Stambaugh, 2017)—i.e., acquisition/training block, retention test, and transfer test—used more controlled and internally valid experimental tasks. On the other hand, investigations of more authentic music performances designed more externally valid musical tasks (for an overview, see Table 4). Finally, a summary of post-hoc results, as described by the authors of the original manuscripts of the studies, is depicted in Table 4, if available. This style of illustration is oriented to Wulf’s (2013) review of FOA effects on motor learning, although the current review does not explicitly focus on FOA effects for the aforementioned reasons. Nevertheless, the results of the small number of included studies convey an impression of how heterogeneous the interplay of participant information, musical tasks, FOA instructions, and OM is (see Tables 1, 4). This fact confirmed the complexity and difficulty of outcome interpretation.

## 4 Discussion

This review aims to overview the current research situation of investigating attentional focus mechanisms in the music performance field by systematically collecting, abstracting, and categorizing relevant data referring to FOA instructions and outcome measures used in studies with experimental paradigms, in accordance with Wulf et al. (1998). In this context, we specifically asked (1) what is the current state of research, (2) what type of FOA instructions and dependent variables are used, and (3) to what extent can a future research agenda be derived from the findings. Out of 387 records initially identified, 15 studies could be included in a more in-depth investigation through several selection steps oriented on the *PRISMA* *statement* (Moher et al., 2009) and the *PRISMA* *2020 item checklist* (Page et al., 2021). Thereby, different types of interesting additional information were collected, that is, aspects of research design, participant information, experimental tasks, and finally, attentional focus effects. Although that data collection was not the main goal of the review, it provides useful information as a basis for the following discussion and supports taking a comprehensive view of the research field and the actual discourse.

### 4.1 What is the goal of movement in a musical performance?

The original FOA paradigm of comparing internal and external foci using their effects on motor learning and performance is well-established in the sports domain and movement science research. Significant results of a superior external FOA can be found in studies examining various types of sports and different kinds of gross motor skills (for reviews and meta-analysis, see Wulf, 2007a; Wulf, 2013; Wulf and Lewthwaite, 2016; Chua et al., 2021). Explanations of these effects, even the specification of distinguishing between proximal and distal external foci, are mainly based on the theory that a shift of attention onto the movement effect or a task goal prevents the constrained action effect and, thus, leads to improved motor performance. With small exceptions, included studies of the current review refer to its research questions, aims (and hypotheses), research designs, experimental paradigms, as well as outcome interpretations to Wulf’s motor theory, and music-specific theoretical constructs, play a marginal role, even during outcome discussions. Overviewing the variety of external FOA instructions in the reviewed studies, the question arises, how a movement goal or movement effect is manifested in music. Many of the authors decided to operationalize the external FOA instructions with wording that targets the *sound* (see Table 1). The challenge with this implementation in the music domain lies in its self-evidence because no musician aims to execute musical movements or sound-producing gestures (Dahl et al., 2010; Jensenius et al., 2010) without aiming to produce sound. Transferring that problem back to the sports domain, we could compare this operationalization with the external FOA instruction to focus on playing basketball or darts (not to shoot baskets, e.g., see Zachry et al., 2005, p. 306; or not to throw bull’s eyes, e.g., see Lohse et al., 2014, p. 124). A few of the included studies from the music domain therefore added some instructions referring to sound *quality* (“thinking about filling the room with their sound,” Atkins, 2017, p. 425; “focus on playing for the audience and the expressive sound of the music,” Mornell and Wulf, 2019, p. 379; “focusing on creating the style of the dance,” Lipke-Perry et al., 2022, p. 425)—as it is a *quality* to shoot baskets in basketball.

### 4.2 Dimensions of musical goals and musical technique

However, these focus-instruction extensions in external FOA music instructions refer to a variety of musical performance dimensions, namely, music communication aspects (“filling the room” or “playing for the audience”), musical expression (“expressive sound”), or musical auditory/visual imagery or musical metaphors (“style of a dance”). These elements of musical performances and musical learning processes could be seen as *musical* goals, as it is not the sound production itself that lies in the musician’s focus of attention, but how the sound is produced, whom the sound is addressed to, and what it should express. Another methodical issue appears when combining different FOA *directions* in *one* instruction. As Wulf herself stated, “[…], we have always attempted to make external and internal focus instructions so similar that they differed in only one or two words to avoid confounds with other variables” (Wulf, 2013, p. 92). Subsequently, she mentioned that “contradictory results” (p. 92)—that is, study outcomes that showed no difference between internal and external FOA or a superiority of internal FOA—could be explained by this aspect. This suggestion of a highly controlled experimental manipulation of FOA instructions was made to optimally trace back FOA effects on motor learning effects to the fact that one word makes the difference. Across all included FOA studies of this systematic review, only two studies are close to transposing this advice (Duke et al., 2011; Stambaugh, 2019). The others more or less failed to control FOA instruction in this regard (see Table 1), at least when paying attention to the number of differently used words.

In addition, there is a big difference between changing words in FOA instructions or consciously referring the instruction to a different musical performance dimension, which took place as well while operationalizing internal FOA instructions. A few of the collected internal FOA instructions relate to technical aspects of musical learning or music performance processes (“focus on the precision of their finger movements (or lip movements for singers) and correct notes,” Mornell and Wulf, 2019, p. 379; “focusing on the fingertips and creating staccato articulation,” Lipke-Perry et al., 2022, p. 4; “focusing their attention on keeping their vibrato steady and consistent,” Atkins, 2017, p. 425). Two points should be discussed concerning aspects of musical technique. On the one hand, the first listed FOA instruction citation refers to the precision of playing (see Mornell and Wulf, 2019). When looking at the control/baseline condition of Stambaugh (2019), she used the wording to play “[…] as accurately as possible” (p. 239), which refers to the same kind of technical dimension. This wording is also used as the basic instruction for all experimental conditions in another study (see Stambaugh, 2017, p. 48). To summarize, we have the same reference of instruction used in three different studies with different functions within the experimental FOA paradigm, i.e., as a control condition, internal FOA, and general instruction underlying all conditions. Reflecting the methodical advice by Wulf (2013) in this context, contradictory results may not be surprising. On the other hand, a technical FOA instruction (“staccato articulation” or “consistent and steady vibrato”) conceptually lacks a precise assignment to one of the two traditional FOAs due to the point that it could be referred to a style of playing or a specific type of sound-production as defined as an external FOA in many studies of this review. Still, all included studies classified technical foci as internal FOA.

### 4.3 FOA in music and multimodal action–perception coupling processes

From a sensory perception perspective, performing music and learning to perform music are processes with many related action–perception coupling mechanisms (Jensenius, 2007; Leman and Naveda, 2010), which are linked with sensory feedback processes as auditory (e.g., Bangert and Altenmüller, 2003; Pfordresher and Chow, 2019), visual (e.g., Wöllner and Williamon, 2007; Bishop and Goebl, 2015), somatosensory (tactile or kinesthetic; e.g., Goebl and Palmer, 2008; Kuchenbuch et al., 2014), or multisensory feedback (for a recent review see Nunes-Silva et al., 2021). In general, musicians spend their whole lives practicing, concentrating consciously or unconsciously on body movements and sound, as the two are inextricably linked. Therefore, it is difficult to argue that a specific focus on one of the two aspects can succeed while not focusing on the other. Furthermore, when it comes to perception, multimodal perception processes, mainly audiovisual integration, are present in making music (see, e.g., Schutz and Lipscomb, 2007). Wulf (2007b) answered a proposal of Hegele and Erlacher (2007) to consider the perceptual dimension of FOA in motor learning, or more concretely, dimensions of the movement effect, with the following statement: The “[…] suggestion that ‘temporal’ and ‘perceptional’ dimensions of movement effects should be considered, […], is interesting. Examining those factors independently would appear to be challenging, however” (Wulf, 2007b, p. 62). She added later: “Nevertheless, examining different dimensions of movement effects would seem like a worthwhile endeavor, as it may provide more insight into the effects of attentional focus on motor control” (p. 62). In the visual perception domain, a few studies (Moore et al., 2012; Klostermann et al., 2014; Rienhoff et al., 2015) investigated the relationship between FOA effects and effects of a visual fixation duration, such as the quiet eye phenomenon (for an overview, see Vickers, 2007; Lebeau et al., 2016). Neugebauer et al. (2020) recently found a significant effect of perceptional-directed attentional foci by implementing a 2 × 2 experimental design using a dart-throwing task (visual–internal vs. visual–external vs. kinesthetic–internal vs. kinesthetic–external), that is, quiet-eye duration was increased in the visual instruction groups. On the other hand, kinesthetic instruction leads to a decrease in visual fixation duration, indicating that the perceptual dimension is highly relevant within FOA research. This outcome is also worthy of discussion because Duke et al. (2011) mentioned this topic from a methodical perspective: “It is important to note that the term focus in this research does not refer to visual focus but to focus of attention (i.e., what one is thinking about). In fact, in much of the laboratory research in this domain, participants look at a visual fixation point throughout all the experimental conditions” (p. 46). To the best of our knowledge, there is just one FOA research attempt to study the auditory perceptual dimension of movement goals in music by Cheng et al. (2011). The group experimentally manipulated the auditory feedback (normal, mute, and delayed)—that was seen by the authors as the external FOA—of a piano task performed by professional pianists. Interestingly, although Cheng et al. (2011) did not use the FOA paradigm by Wulf et al. (1998), they had no verbal FOA instructions. Instead, they apparently pre-supposed that the sound of the piano *is* the movement goal of the pianists, and the absence or manipulation of fingering while playing was associated with the presence or the absence of an internal focus. Nevertheless, the results show that even when the fingering was manipulated on instruction, the most important performance factor was auditory feedback. Based on this outcome, it is worthwhile to examine the relationships between auditory or audiovisual sensory feedback and FOA effects in music.

### 4.4 Musical performance outcomes: measuring more or less?

One of the core aims of the current review was to shed light on the type of application of outcome measures possible within FOA research in the music performance domain. We, therefore, collected and categorized all data of dependent variables used to measure FOA effects on the accomplishment of the different musical tasks. As already mentioned, the number of different kinds of outcome measures was surprisingly high in relation to the small number of studies that met the eligibility criteria of inclusion and compared to the amount of applied OM in many motor learning studies from the sports domain (see Wulf, 2013; Chua et al., 2021). It almost seems as if many studies reviewed in this article used several dependent measures to find the effects of experimentally manipulated FOA instructions. One of the reasons for that high amount of OM could lie in the dimension of the movement goal in music, the *sound*. Identifying *sound* as the external FOA means finding a solution to measure the performance effect, and this challenge was solved partially by conducting an acoustical analysis (Atkins and Duke, 2013; Atkins, 2018; Allingham et al., 2021; Allingham and Wöllner, 2022; see Table 4). Considering the amount of existing information of (post-hoc) results within the original manuscripts (see Table 4) of the studies, the question arises, how exploratory in its origin research designs of FOA studies in music are. Directed hypotheses are rare and, if available, a theoretically and/or empirically derived answer to why the experimental manipulation should affect this certain outcome measure, if at all, is slightly posed. Vice versa, the methodical sharpness of the FOA paradigm in its original implementation in motor learning research—and the pervasive number of significant results supporting the superiority of adopting an external FOA—provides a solid base for a precisely formulated, directed hypothesis. We argue that one of the key aspects of this methodical issue or challenge of analysis selection is a missing theoretical underpinning of FOA effects in music in general. If we do not know how it works, we know even less how to measure it. Another issue lies in the general challenge of measuring musical performance (see Schmidt and Lee, 2012). Expert ratings have been established through their extensive use within different music educational settings, grading in the music study context, performance evaluations in music competitions, and judgments with scores for various musical performance characteristics in auditions for positions as professional musicians. However, the high amount of different OMs used in the growing FOA music field somehow prevents comparable results. Thus, the interpretation of outcomes is challenging. Moreover, this problem is grounded on the inconsistency of applied musical tasks and the musical instruments used in the studies (see Table 4). Singers, wind instrument players, and pianists have different motor skills, different sensory awareness, gestural flexibility, and finally, a different perceptual-directed focus of attention during musical performance. There is a lack of replication studies and a lack of studies with different subjective performance ratings, such as ratings of competitors or the audience, which play a big part in the discourse of musical goals.

### 4.5 Limitations of the review study

The limitations of the review study are mainly due to the small number of experimental studies. In addition, the inconsistent application of analysis between those studies leads to problems in classifying the results and reported effects. Challenges to the interpretation of study results from the FOA in the music field impede formulations of credible insights. Furthermore, it is not possible to estimate the effects of internal, external, or otherwise different attentional focus instructions on musical learning, music performance, or motor learning in music based on this investigation. Another limitation lies in various steps of the *PRISMA guidelines* to conduct and display systematic reviews, which are in their implementation—even if the claim is to be as transparent and objective as possible—inherently still subjective. We tried to strictly follow the checklist and explain why we deviated from it in some places. Moreover, data abstraction, data categorization, and the design of the categorization systems in terms of FOA instructions and outcome measures are explorative in nature. However, the specific goal of this review lies in focusing on the methodical aspects in detail and not evaluating the results and effects of the reviewed studies. Transparent elucidations of conducting this inductive-deductive style of analysis were given so they can be discussed or criticized in the field and may serve as an impulse for further investigations in this regard. Finally, the definition of eligibility criteria in systematic reviews is often challenging as it determines the study selection process. Formulations of the eight criteria are somehow worthy of discussion, e.g., the requirement that studies must apply an experimental FOA paradigm in reference to the Wulf et al. (1998) attempt (criteria e) and contain a precise description of FOA instruction (criteria g). There are a few theoretical contributions overviewing the situation of FOA research in singing (e.g., Helding, 2015, 2016; Brand, 2021; Treinkman, 2021, 2022b), but they failed these criteria. Other studies used qualitative methods to provide important ideas on studying FOA in music (e.g., Buma et al., 2015; Guss-West and Wulf, 2016; Oudejans et al., 2017; Parsons and Simmons, 2021; Treinkman, 2022a; Lubert et al., 2023); however, they were not included due to missing criteria (e) and (g). The study by Cheng et al. (2011); published in conference proceedings and a few unpublished dissertations (e.g., Atkins, 2013; Mentzel, 2016; Williams, 2019; Allingham, 2022) approaching aspects of FOA in music were excluded—besides other reasons—because they failed criteria (c), i.e., reports were not published in a peer-reviewed journal or we could not finally be sure of it. With the exception of the study by Mentzel (2016), the mentioned unpublished dissertations described the same studies that were included in the review. Our decision to apply somehow strict criteria in this regard is grounded on the aim of deepening face up to two of the relevant aspects of the discussion, namely FOA instructions and outcome measures.

### 4.6 Implications and future studies

Almost every discussion or conclusion section of the study reports included in this review contains ideas of music pedagogical implications from their study results. In other cases, the authors have concrete suggestions of didactical implications for practicing music in general or, in more detail, for singing and playing an instrument. Considering the inconclusive results and effects of FOA instructions on music performance, we would not make concrete music pedagogical suggestions for using shifts of FOA while practicing music at this point. One of the main reasons for that is the absence of FOA studies in music with a learning paradigm in an application-oriented educational setting. Concerning on-stage musical performances in *performance under pressure* contexts, the research situation manifests as tighter due to a long tradition of multidisciplinary investigations in relation to anxiety and stress in high-pressure performing settings from psychology, sports, and music, e.g., the growing field of music performance anxiety research (for current reviews, see Fernholz et al., 2019; Osbourne and Kirsner, 2022; Kenny, 2023). However, studies showed that dealing with music-specific high-pressure challenges is highly individual and diverse, and depends on multiple factors (see, e.g., Buma et al., 2015; Oudejans et al., 2017). When dealing with the wide range of practice routines at different practice stages (see Antonini Philippe et al., 2020) and the very different pedagogical approaches of instrumental and vocal teachers, it is very difficult to assess the impact of conscious changes in attentional focus on musical learning. This is also supported by anecdotic evidence from discussions with many music students in different lectures, where practice and teaching routines, the practice stage, and the musical literature play a major role.

In relation to the last-mentioned issue and as a preliminary conclusion, future studies of FOA effects in the music domain should first go a step back. For the construction of a music-specific theoretical model, it is necessary to take the musical goals of musicians seriously. Undoubtedly, basic principles of motor learning and motor control have an important influence on how successful musicians learn and perform, but when supposing an integral understanding of musical goals, mental and physiological health and wellbeing should be noted as well. We would argue that it is necessary to conduct big qualitative and quantitative questionnaire surveys to collect as much as possible of existing FOA routines and pedagogical instructions in various music-relevant learning and performance situations of musicians with different goals. Subsequently, it could be interesting to do some in-depth investigations of singular practicing and on-stage performances, e.g., by applying video-stimulated-recall settings (see, e.g., Després, 2022), in which the relation between conscious or involuntary shifts of attentional foci, musical material, and sound characteristics could be analyzed solidly. Maybe a strict inductive strategy of data analysis will generate clusters, categories, or structures that somehow confirm the reasonableness of a transfer of the original binary FOA paradigm by Wulf et al. (1998) from sports to music—maybe not, however, because dimensions of musical performances could be more complex.

## 5 Conclusion

To conclude the current review, we start with a reference to the three main research questions. Out of 387 records, a small number, 15 studies, were included in the study, which, by definition of certain eligibility criteria, applied a FOA instruction paradigm in an experimental design. FOA instructions could be abstracted into 10 different subcategories, e.g., *sound focus*, *visual focus*, *bodily focus,* or *instrumental focus*, which again could be assigned to the two classifications of internal and external FOAs. We classified 63 outcome measures into 9 outcome measure categories, e.g., *acoustical analysis* and *expert rating*. Future scientific discourse in the field should focus on exploring musical goals as one of the critical aspects when comparing FOA effects in sports with those that could be expected in music. We could show that the current research situation is lacking in various points that must be considered before generalizing insights and offering music pedagogical implications. Finally, it seems to be promising to pursue a new application-oriented attempt at exploratory research on attentional focus routines and FOA shifts of musicians in different practice stages to get a solid database of music-specific attentional foci.

## Data availability statement

The datasets for this study can be found in the Supplementary material (see Review protocol).

## Author contributions

JH: Conceptualization, Investigation, Methodology, Visualization, Writing – original draft, Writing – review & editing. AI: Conceptualization, Investigation, Methodology, Visualization, Writing – review & editing.
